# Blink rate as a measure of stress and attention in the domestic horse (*Equus caballus*)

**DOI:** 10.1038/s41598-020-78386-z

**Published:** 2020-12-08

**Authors:** Richard O. Mott, Susan J. Hawthorne, Sebastian D. McBride

**Affiliations:** 1 The Royal (Dick) School of Veterinary Studies, The University of Edinburgh, Midlothian, UK; 2 School of Pharmacy and Pharmaceutical Sciences, Ulster University, Coleraine, Co. Londonderry UK; 3 Aberystwyth University, Penglais, Aberystwyth, Ceredigion UK

**Keywords:** Neuroscience, Physiology, Zoology

## Abstract

Measuring animal stress is fundamentally important for assessing animal emotional state and welfare. Conventional methods of quantifying stress (cortisol levels, heart rate/heart rate variability) require specialist equipment and are not instantly available. Spontaneous blink rate (SBR) has previously been used to measure stress responses in humans and may provide a non-invasive method for measuring stress in other animal species. Here we investigated the use of SBR as a measure of stress in the domestic horse. SBR was measured before and during a low-stress event (sham clipping) and compared with heart rate variability and salivary cortisol. For the entire sample, there was a reduction in SBR (startle response) during the first minute of clipping. For horses reactive to clipping, the initial reduction in SBR was followed by an increase above baseline whereas the SBR of the non-reactive horses quickly returned to baseline. For the entire sample, SBR correlated with heart rate variability and salivary cortisol. We have demonstrated that SBR is a valid fast alternative measure of stress in horses, but the initial 'startle' response must be considered when using this parameter as a measure of animal stress.

## Introduction

Measuring animal stress is fundamentally important to ensuring reasonable levels of animal welfare^1^. Conventional methods of quantifying stress include measuring plasma and saliva cortisol levels^2–4^, eye temperature^5,6^, heart rate and heart rate variability^7–9^. The primary disadvantage associated with these methods is that they require specialist equipment and training in how to collect and/or analyse the data. The results are also not instantly available, requiring at least some degree of processing and analysis. Spontaneous blink rate (SBR) has previously been used to measure stress responses in humans and may provide a non-invasive method for measuring stress in a range of species^10^. Spontaneous blinks have several functions, they are essential for corneal lubrication and also appear to be important for resetting eye movements during the correction of fixation errors ^11^. Blinks also reflect cognitive processes during attention, with blink rate increasing when attentional demand is low (e.g. during punctuation pauses whilst reading^12^), during pauses while listening to a speech^13^ and also during information retrieval and memory^14,15^. The link between SBR and stress appears to be mediated via dopaminergic activity. SBR significantly increases in monkeys through the sub-cutaneous injection of dopamine agonists (apomorphine) and significantly blocked with dopamine antagonists (sulpiride)^16^ and CNS dopamine levels significantly increase during stressful events. The latter reflects this neurotransmitter’s functional role in eliciting motivation and learning processes during homeostatic behavioural responses to stress^17,18^.

Although the majority of evidence demonstrates that SBR increases during stress^19,20^ other work in horses has reported a decrease in SBR when presented with a potentially stressful event^21^. Human studies have shown a reduction in SBR during periods of attention and cognitive effort^20,22–25^. Potentially threatening stimuli may require cognitive effort to verify it as a threat thus reducing SBR at the early stage of stressor presentation. This suggests that the SBR response to stress could vary depending on (1) when it is measured in relation to the stressor exposure and (2) the type of stressor. Using the horse as a model species, the aim of this study was twofold, firstly, to validate SBR as a putative measure of stress against conventional measures of stress (HRV and salivary cortisol), and secondly, to assess the timing and consistency of the SBR response during a stress event.

## Results

### Spontaneous blink rates

**Figure 1:**
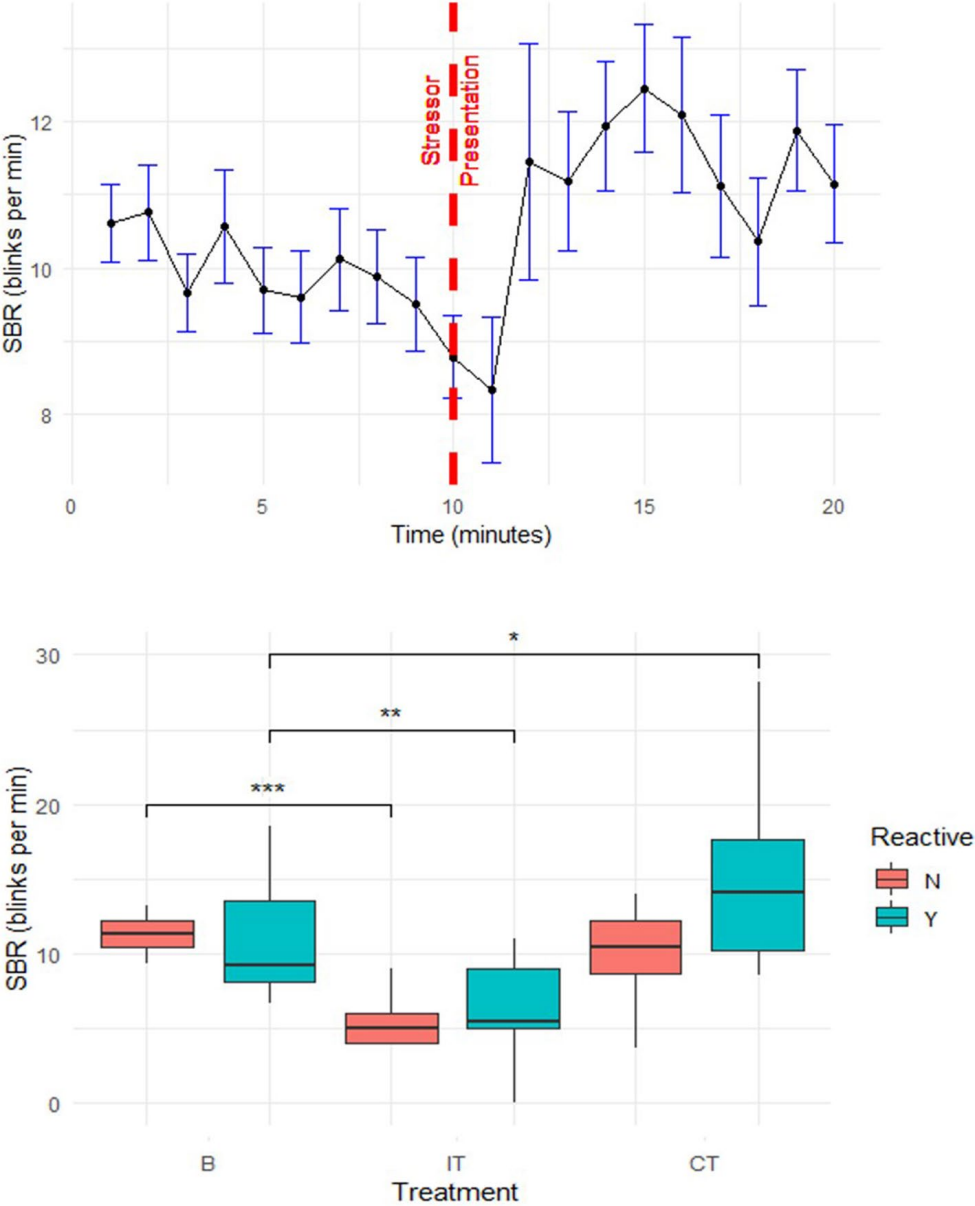
SBR over time for reactive and non-reactive horses. Mean SBR (SEM) (blinks min^−1^) for reactive and non-reactive horses (n = 33) during baseline (B) (minutes 1–10), initial treatment (IT) (minute 10, 1 min post-stressor presentation) and continued treatment (CT) (minutes 11–20 post-stressor presentation). P < 0.05 (*), P < 0.01 (**), P < 0.001 (***).

Figure 1 shows the mean SBR (± SEM) over time with the stressor presented at the 10 min point and maintained thereafter for the duration of the experiment.

When partitioned into low (n = 16) and high (n = 17) reactive groups, the high reactive group showed a highly significant decrease in SBR during the IT period compared to baseline (11.1 ± 1.09 blinks per min to 6.2 ± 0.95 blinks per min, t_1,30_ = − 2.9, p = 0.008) and a significant increase in SBR from baseline during the CT period (11.1 ± 1.09 blinks per min to 15.6 ± 1.71 blinks per min, t_1,30_ = 2.7, p = 0.012). For the low reactive group, there was also a highly significant decrease in SBR during the IT compared to baseline, (11.4 ± 0.33 blinks per min to 5.8 ± 0.46 blinks per min, t_1,32_ = − 7.9, p < 0.001), but no significant difference in SBR during CT compared to baseline B (11.4 ± 0.33 blinks per min to 10.0 ± 0.67 blinks per min, t_1,32_ = − 1.9, p = 0.068).

### Correlation of SBR with standard measures of stress

**Figure 2:**
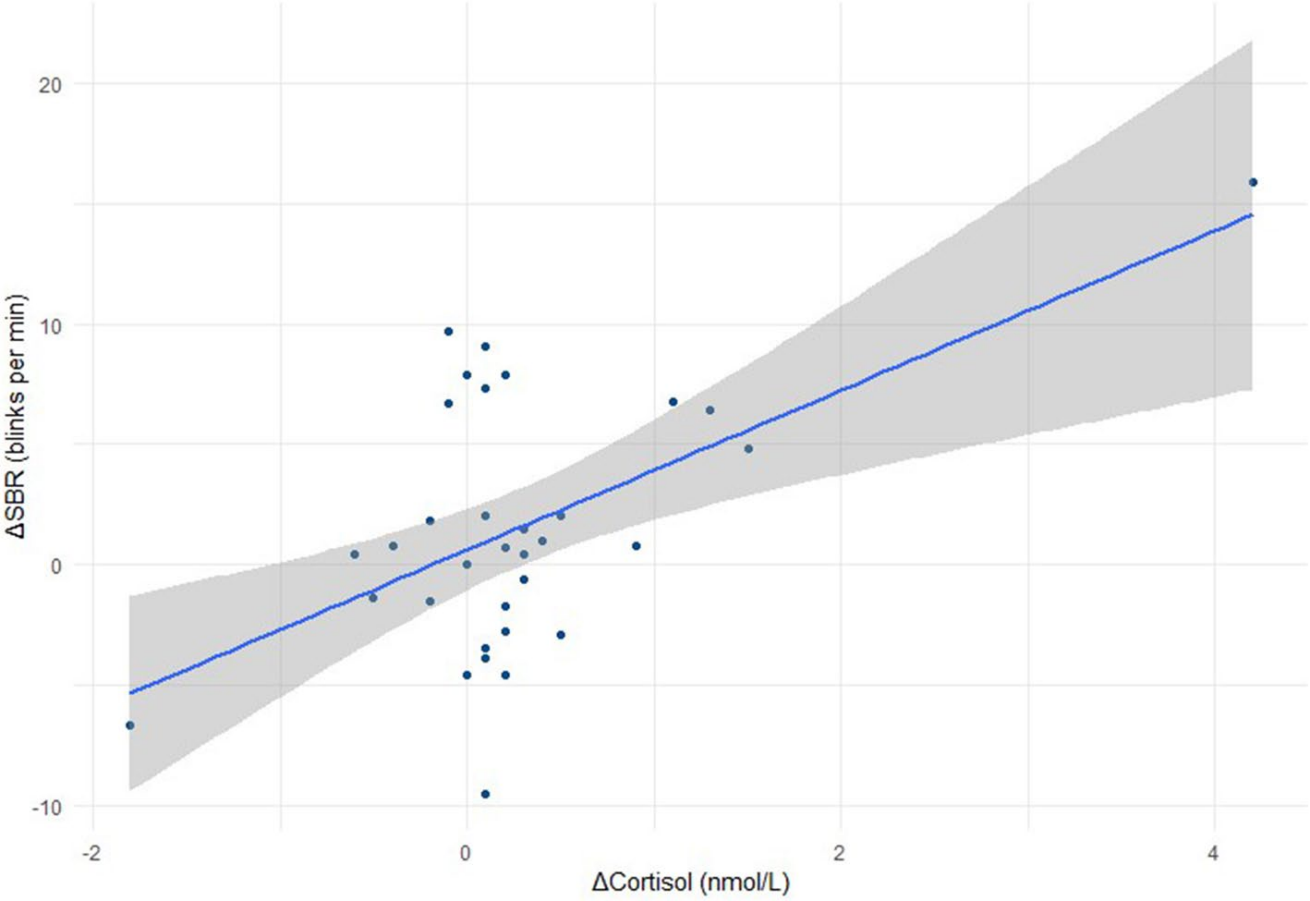
Correlation of SBR with Cortisol. Correlation of change in SBR (B verses CT) against change in Cortisol (B verses CT) for all horses (low and high reactive; n = 33) (r = 0.56, p < 0.001).

**Figure 3:**
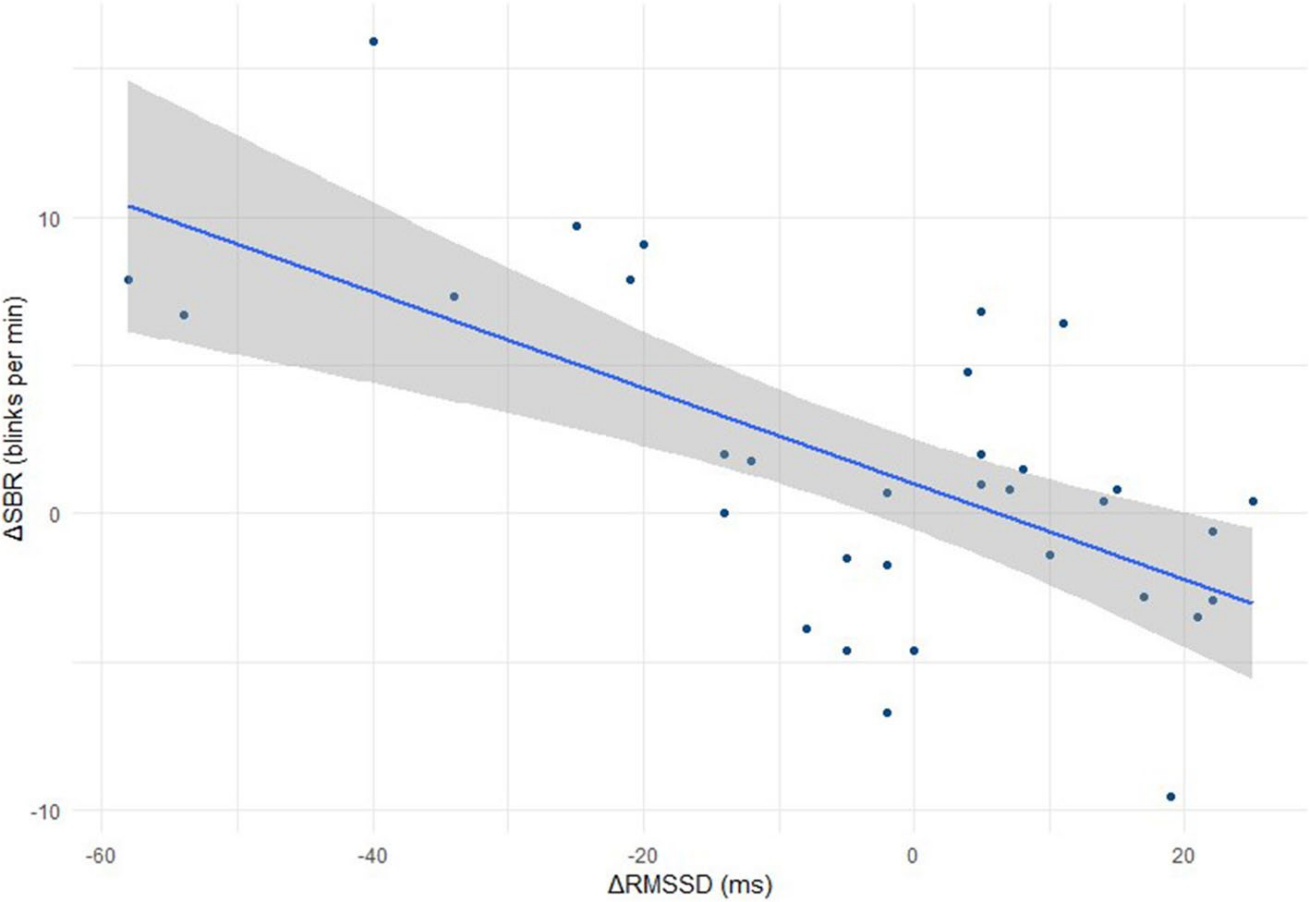
Correlation of SBR with RMSSD. Correlation of change in SBR (B verses CT) against change in RMSSD (B verses CT) for all horses (low and high reactive; n = 33) (r = − 0.63, p < 0.001).

For all animals (low reactive and high reactive) combined, there was a highly significant moderate positive correlation between the change in SBR between baseline (B) and the continued treatment (CT) (ΔSBR) and change in cortisol (ΔCortisol) (r = 0.56, p < 0.001) (Fig. 2) and a highly significant strong negative correlation between ΔSBR and ΔRMSSD (r = − 0.63, p < 0.001) (Fig. 3).

The HRV and cortisol results for each horse at each time-point can be found in Supplementary Table S1 online.

## Discussion

A stress response was seen in both the HPA axis and SAM system demonstrating a combined axes response to a psychological stressor. It has been suggested that activation of the SAM axis predominates during times of physical stress whereas the HPA axis is activated during psychological stress^26–28^. The results presented here demonstrate that a predominantly psychological stressor elicits a response in both the HPA and SAM axes, with significant elevation in saliva cortisol and reduction in heart rate variability respectively.

The increased SBR correlating with an increased physiological stress response agrees with previous studies in humans^19,20^ but conflicts with the previous study in horses^21^ that reported a reduced SBR during stressful situations. One of the key differences between the work of Merkies et al.^21^ and the present study is that the former used three stressors, one of which was a simple startle test and the other two (separation and withholding food) were only recorded for the first 3 min. Here we report that during the first minute of the stressor (sham clipping), for both low and high reactive animals, there was a highly significant reduction in SBR. The reduction was larger in the non-reactive group and from the video analysis it could be seen that many of the high reactive horses had a very distinct period of non-blinking/startle response at the initial presentation of the clippers, but this only lasted a few seconds before rapidly increasing. As previously discussed, the initial SBR response may reflect an initial increase in concentration/vigilance response to the stressor^20,22,23^ which would explain why in the early stages of a stressful event (particularly a startle test), a reduced SBR was observed^21^.

It was of note that low reactive horses (that did not become stressed by the sham clipping stressor event) spent longer in a state of low SBR-high concentration response (1 min or more verses a few seconds for the highly reactive group). This strongly suggests that more stress reactive animals may also be prone to making faster environmental assessments in relation to potential threats. However, it must also be considered that none of the animals in the current study were naïve to the stressor, so for highly reactive horses, this may also have been a learned response that did not need lengthy consideration. Overall, the data demonstrate that the initial startle response, as shown by the initial significant reduction in SBR, needs to be taken into consideration when using SBR as potential measure of animal stress.

After the first minute following stressor presentation and the startle response, high reactive horses (n = 16) produced a highly significant increase in SBR above baseline, which was maintained for the duration of the experiment, whilst low reactive animals' SBR returned to baseline levels after the first minute. SBR therefore appeared to be a valid parameter of stress measurement. One of the primary aims of this study was to validate SBR as a measure of stress through correlation against conventional measures of stress centred around activation of the HPA and SAM axes. The Pearson’s correlation between ΔSBR and the change in stress parameters for the whole sample (n = 33), found a highly significant (p ≤ 0.001) correlation between ΔSBR and HRV (as a measure of SAM system activation—more specifically, RMSSD as an implied measure of parasympathetic/vagal tone) and salivary cortisol (as a measure of HPA axis activation), with a higher SBR correlating with a higher physiological stress response. The correlation between SBR and RMSSD was marginally stronger (0.63) than the correlation between SBR and salivary cortisol (0.53). This may suggest that SBR reflects greater activation of one axis over the other. SBR is mediated by activation of the D1 and D2 dopamine receptors in the striatum^16^ and stress increases levels of dopamine (DA) in this region of the brain^17,18,29,30^. Although there are glucocorticoid receptors at both ends of the messoaccumbens pathway (ventral tegmentum area and nucleus accumbens) and increased levels of glucocorticoids initiate DA release along this mesoaccumbens pathway^31^, there is also a temporal lag (approximately 30 min) between stressor exposure and peak plasma glucocorticoid levels^2–4,26^. Thus, the release of glucocorticoids into the peripheral blood system to then stimulate glucocorticoid receptors in the striatum cannot be attributed as the causal factor for increased SBR. However, Corticotropin Releasing Hormone (CRH) (which initiates the neuroendocrine signalling in the HPA axis) can also stimulate DA release in the ventral striatum^32^ suggesting that the SBR may be mechanistically related to the co-activation of the HPA axis.

In relation to the SAM axis, the sympathetic nervous system (SNS) is activated during periods of high vigilance^33^ and thus could be responsible for the reduced SBR reported during the IT phase in this and other studies^21,34^. However, dopaminergic innervation of the central nucleus of the amygdala from substantia nigra appears to be critical for the enhanced attention response during presentation of unexpected stimuli via its control over the parietal cortex^35^. It is well established that the posterior parietal cortex is a key structure during attentional response and mediates this functional role through direct influence over the frontal eye fields and superior colliculus to control volitional eye movement^36^ as well as blink rate^37^. This may therefore be the primary candidate mechanism underlying both the reduced blink rate during the startle response and the heightened blink rate during more prolonged periods of stress-inducing elevation of central dopaminergic systems.

A limitation of the study is that we were unable to specifically quantify the SNS activity of the ANS. Previously, this has been assumed from measures of LF Power and LF/HF ratio of HRV, but this method has been widely criticised^38–40^. As outlined in the review by Stucke, et al.^41^, it would be inappropriate to draw any inference about SNS activity from the time domain parameter reported in this study, although this does not detract from the application of SBR as a welfare assessment tool. Measurement of electrodermal activity (EDA) is a renowned marker of sympathetic activity^40^ and use of this measure may further elucidate the relationship of the autonomic nervous system in the blink response to stress.

## Conclusion

SBR is a simple, cost-free, instantaneous measure of acute equine stress that can support welfare assessment, as demonstrated through the significant increase from baseline during exposure to a known stressor. This is further validated through the significant correlation of SBR with conventional measures of stress associated with the activation of the HPA and SAM axes. The startle response must be taken into consideration when using SBR as a measure of stress and the duration of the startle response may vary significantly between low and high stress reactivity animals. As a final point and caveat to the use of SBR as a measure of animal stress, dopamine also increases in the striatum during the presentation of reward substrates^42^. Thus theoretically, reward or positive events may also induce a significant increase in SBR. Understanding the applied use of the SBR response, both in horses and other animal species, will be improved by future research assessing the effects of positive stimuli on changes in SBR and also further validation of SBR against conventional measures of stress based around the SNS and HPA axis.

## Methods

### Animals

Thirty three horses (mean ± SEM age 13 ± 0.7 years; 21 geldings, 12 mares) stabled at the Moreton Morrell campus of Warwickshire College were used for the study. All horses were physically fit in regular exercise as general riding school horses for up to 4 h per day and none were on any medication during sampling or for the 2 weeks prior to sampling. Diets were individually tailored depending on size and workload but generally the dietary intake was divided 70–80% haylage, 20–30% cool mix (9.8 MJ/kg / 10% protein). All horses were housed in an American barn style stable block. The upper half of the stable partitions were bars, allowing the horses to see conspecifics on either side of them as well as those opposite. Four hours of group turn-out was available every other day. Of the 33 horses used in the study, 16 were categorised by animal staff as reactive to the exposed stressor (sight and sound of hair clippers) (high reactive) and the 17 remaining animals were categorised non-reactive to the stressor (low reactive). This assessment was based on the horses’ previous reactions to being clipped and human safety during the clipping process (in their role as teaching horses at the equine college); the 17 non-reactive horses were designated as safe for students to clip, whereas the 16 reactive horses required a member of staff to clip them or had to be sedated during the clipping process. Ethical approval for this study was granted by the Veterinary Ethical Review Committee for the Royal (Dick) School of Veterinary Studies and the Research Ethics Committee for Warwickshire College Group. All methods were performed in accordance with the guidelines and regulations of these two institutions.

### Study design

Data were collected during a period of experimentally-induced mild stress following a protocol similar to Yarnell et al.^6^, whereby horses were subjected to a controlled ‘sham clipping’ (sight and sound of hair clippers) for a period of 10 min. This entailed holding a set of clippers (Liveryman Arena C130, UK) against the horses’ left shoulder in a way that the hair was not actually cut. Ten minutes of clipper exposure has previously been reported to cause a significant elevation in salivary cortisol^6^ and thus this same duration was used in this experiment. Each horse acted as its own control to assess the relationship between variables (within-horse) during periods of stress and no stress, with measurements taken during the sham clipping being compared with the ‘at rest’ measurements for that horse. The ‘at rest’ measurements were always taken before exposure to the stressor in order to avoid any carry-over effects and each horse was only sampled once. All measurements were taken between 1100 and 1300 each day to avoid any confounding influences of diurnal variation in physiological recordings^43^. Each procedure was carried out in the horses’ own stable.

### Heart rate variability

Each horse was fitted with a heart rate monitor (Polar Equine V800 Science, Polar Electro Oy, Kempele, Finland) and given a 5 min acclimatisation period. Heart rate and video recording was then initiated at the same time to ensure that the time stamp on the video would relate directly to the time on the HR trace. The horse was monitored for 10 min at rest before the clippers were started and the sham clipping procedure conducted for the next 10 min. This provided a continuous 20 min video and HR trace.

Heart Rate Variability (HRV) time domain analysis (Root mean square of the successive differences of the beat to beat interval, recorded in milliseconds [RMSSD]) of HRV data were sampled for 10 min using a Polar Equine V800 Science heart rate monitor. The earlier model (Polar S810) has been validated in horses^44,45^ and although Parker et al.^44^ found discrepancies existed during movement, both Parker et al.^44^ and Ille et al.^45^ found better agreement when the horse was static (as they were during the current study). Randle et al.^46^ proposed that recent technological improvements (i.e. the newer Polar V800) could improve the reliability of the data generated and Giles et al.^47^ demonstrated that (in humans) the V800 was indeed an improvement over the earlier models. Although this specific model has not been validated in horses, it has demonstrated good interclass correlations with a clinical ECG in humans^47^.

RMSSD and SDNN are both measures of PNS activation, but SDNN is a more suitable metric for long-term changes (minimum of 5 min required)^39^. SD1 is identical to RMSSD^48^, although some authors still report these as separate measures. SD2 closely correlates to SDNN and is therefore only suitable for assessing long-term variance. We initially anticipated reporting the LF/HF ratio as cautiously suggested in the review by Stucke et al.^41^ as a measure of SNS activity in horses, but this (and LF power) are now seen as unreliable measures of SNS activity (in humans)^40^. There is president in applying the findings of human studies to other mammals as outlined in the review by Von Borell et al.^9^, primarily because the neural basis of an emotional response is similar in all mammals, as is the control of vagal tone. It could be argued that the uniquely high resting vagal tone of horses makes them a special case^41^, but in the interests of robustness, the LF/HF ratio and LF Power where excluded from this analysis.

Data were analysed using Kubios software (Kubios HRV Standard version 3.1.0, Kubios Oy, Kuopio, Finland^49^), using a low threshold (0.35 s) for artifact correction. Any data with more than 5% errors were discarded^50^.

### Salivary cortisol

Salivary cortisol samples were taken whilst the horse was at rest 15 min prior to the start of recording and again at 60 min after the start of the clipping procedure. This took into account the time lag of the peak increase in salivary cortisol following a stressful event^2,3^ and avoided sampling (itself a potential stressor) during the recording period.

Saliva samples were taken using Salivette cortisol swabs (Sarstedt, Nümbrecht, Germany). The swabs were mounted in Foerster 9.5 inch straight sponge forceps to avoid accidental loss and to ensure the safety of the operator. Using these forceps, the swab was held under the horses’ tongue for 90 s. In accordance with the manufactures’ instructions, the Salivettes were stored at 4 °C immediately after collection and then centrifuged at 1000 g for 2 min (Sigma 3K30 Centrifuge, Sigma Laboratory Centrifuges, Osterode am Harz, Germany). The extracted saliva was frozen in the Salivette collection tube at − 20 °C within 1 h of collection. The frozen samples were then transported, on dry ice, for analysis within 3 months of collection. The cortisol competitive immunoassay was carried out using a Salimetrics salivary cortisol enzyme immunoassay kit (Stratech Scientific Ltd., Ely, UK) in accordance with the manufacturer’s instructions.

Both the Sarstedt swabs and the Salimetrics ELISA have been previously been validated for use in the horse^2,3,51,52^.

### Spontaneous blink rate

The method for measuring SBR in horses has previously been described by Roberts et al.^53^. In brief, the horses were lightly restrained using a head-collar and lead-rope to prevent them from moving around and observations were made by a single observer with a clear view of the horses’ left eye. Only one eye can be observed at any one time due to their lateral positioning. The left eye was selected following the experimental protocol adopted by Roberts et al.^53^ and also because horses demonstrate a left gaze bias in relation to stressful events^54^. Thus, measuring the left eye was considered to give a more accurate indication of the stress response and was kept consistent throughout the project. Furthermore, it is convention to approach and handle horses on their left-hand side, so filming from the left was potentially less likely to cause a stress response than if the horses’ head was approached from the right-hand side.

The area of the eye was filmed using a High Definition digital camera (Fujifilm Finepix S6800) at 60 frames s^−1^ to allow post hoc measurements to be made. Blink rate was reported as blinks per minute by the same observer for each sample. In contrast to the reporting of eye flutters and half closures by Merkies et al.^21^, this study counted the binary eye fully open or eye fully closed. As this was not a subjective measure, it was felt that the addition of a second, blinded observer would have only offered a minimal improvement in reporting. A clip of the video collected can be found in the Supplementary Information online.

### Statistical analysis

All statistical analysis and data visualisation were carried out using R version 3.5.3^55^.

A linear mixed effects model (with individual horse as the random effect) was fitted to the data to compare the SBR at baseline (B) (minutes 0–10) with the initial treatment (IT) (minute 10) and with the continued treatment (CT) (minutes 11–20). The model assumptions were tested by plotting the normal probability of the residuals. The change in each parameter was calculated (during stress minus before stress) and then all the parameters were compared using a Pearson Correlation. When performing the correlations, the data were analysed as the complete sample (N = 33). For analysis of the SBR, the data were partitioned into the two groups (low reactive [N = 16] and high reactive [N = 17]).

## Supplementary information

Supplementary Information 1.

Supplementary Information 2.
