# Supplementary material for: Blink rate as a measure of stress and attention in the domestic horse (Equus caballus)
Source: Sci Rep. 2020 Dec 8;10:21409. doi: 10.1038/s41598-020-78386-z (PMC7722727; doi:10.1038/s41598-020-78386-z)
Supplement: Supplementary file 1 — Supplementary Information 1. [file 41598_2020_78386_MOESM1_ESM.docx]

Supplementary Data for

**Blink rate as a measure of stress and attention in the domestic horse (*Equus caballus*)**

Richard O Mott^1^*, Susan J Hawthorne^2^ and Sebastian D McBride^3^

1. *The Royal (Dick) School of Veterinary Studies, The University of Edinburgh, Midlothian, UK*
2. *School of Pharmacy & Pharmaceutical Sciences, Ulster University, Coleraine, Co. Londonderry, UK*
3. *Aberystwyth University, Penglais, Aberystwyth, Ceredigion, UK*

*****Corresponding author: [Richard.Mott@glasgow.ac.uk](mailto:Richard.Mott@glasgow.ac.uk)

Table S1: Physiological measures for each horse at each time-point.

| **Horse** | **Time Point** | **Mean SBR** | **HR** | **RMSSD** | **Cortisol** |
| --- | --- | --- | --- | --- | --- |
| **Number** | **B/IT/CT** | **blinks min^-1^** | **bpm** | **ms** | **nmol/L** |
| 1 | B | 18.5 | 32 | 95 | 1.3 |
| 1 | IT | 6 | 70 | 77 | NA |
| 1 | CT | 26.4 | 43 | 74 | 1.3 |
| 2 | B | 9.2 | 26 | 67 | 2.1 |
| 2 | IT | 0 | 31 | 129 | NA |
| 2 | CT | 14 | 26 | 71 | 3.6 |
| 3 | B | 11.2 | 37 | 31 | 1.1 |
| 3 | IT | 4 | 48 | 43 | NA |
| 3 | CT | 12.2 | 41 | 36 | 1.5 |
| 4 | B | 13.2 | 30 | 52 | 1.1 |
| 4 | IT | 6 | 35 | 90 | NA |
| 4 | CT | 3.7 | 31 | 71 | 1.2 |
| 5 | B | 6.9 | 35 | 125 | 1.1 |
| 5 | IT | 11 | 51 | 115 | NA |
| 5 | CT | 13.6 | 36 | 71 | 1 |
| 6 | B | 8.6 | 32 | 73 | 0.7 |
| 6 | IT | 9 | 33 | 135 | NA |
| 6 | CT | 8.6 | 31 | 59 | 0.7 |
| 7 | B | 9.3 | 31 | 54 | 1.4 |
| 7 | IT | 4 | 58 | 55 | NA |
| 7 | CT | 7.9 | 43 | 64 | 0.9 |
| 8 | B | 10.4 | 27 | 51 | 1 |
| 8 | IT | 9 | 36 | 56 | NA |
| 8 | CT | 7.5 | 27 | 73 | 1.5 |
| 9 | B | 12.2 | 24 | 92 | 5.5 |
| 9 | IT | 5 | 24 | 69 | NA |
| 9 | CT | 5.5 | 24 | 90 | 3.7 |
| 10 | B | 13.6 | 25 | 82 | 1.3 |
| 10 | IT | 5 | 26 | 112 | NA |
| 10 | CT | 9 | 24 | 77 | 1.3 |
| 11 | B | 13.2 | 38 | 31 | 1.5 |
| 11 | IT | 5 | 45 | 42 | NA |
| 11 | CT | 14 | 42 | 38 | 2.4 |
| 12 | B | 6.7 | 35 | 103 | 1.7 |
| 12 | IT | 5 | 57 | 97 | NA |
| 12 | CT | 22.6 | 46 | 63 | 5.9 |
| 13 | B | 12.4 | 31 | 69 | 1.6 |
| 13 | IT | 6 | 37 | 118 | NA |
| 13 | CT | 13.1 | 29 | 67 | 1.8 |
| 14 | B | 12.2 | 24 | 93 | 3.5 |
| 14 | IT | 5 | 25 | 70 | NA |
| 14 | CT | 10.5 | 24 | 91 | 3.7 |
| 15 | B | 8.6 | 32 | 72 | 0.7 |
| 15 | IT | 9 | 36 | 138 | NA |
| 15 | CT | 10.6 | 33 | 58 | 0.8 |
| 16 | B | 6.9 | 35 | 127 | 1.1 |
| 16 | IT | 11 | 52 | 113 | NA |
| 16 | CT | 14.8 | 43 | 69 | 1.3 |
| 17 | B | 18.5 | 32 | 94 | 1.2 |
| 17 | IT | 5 | 71 | 78 | NA |
| 17 | CT | 27.6 | 45 | 74 | 1.3 |
| 18 | B | 9.2 | 26 | 68 | 2.3 |
| 18 | IT | 0 | 38 | 132 | NA |
| 18 | CT | 16 | 28 | 73 | 3.4 |
| 19 | B | 11.2 | 36 | 33 | 1.1 |
| 19 | IT | 4 | 47 | 45 | NA |
| 19 | CT | 13.2 | 41 | 38 | 1.6 |
| 20 | B | 9.3 | 31 | 52 | 1.2 |
| 20 | IT | 4 | 57 | 55 | NA |
| 20 | CT | 10.1 | 43 | 67 | 0.8 |
| 21 | B | 13.6 | 25 | 79 | 1.3 |
| 21 | IT | 5 | 27 | 103 | NA |
| 21 | CT | 9 | 25 | 79 | 1.5 |
| 22 | B | 11.3 | 35 | 30 | 1.2 |
| 22 | IT | 4 | 49 | 41 | NA |
| 22 | CT | 12.8 | 41 | 38 | 1.5 |
| 23 | B | 12.2 | 34 | 96 | 3.4 |
| 23 | IT | 8 | 36 | 73 | NA |
| 23 | CT | 10.7 | 34 | 91 | 3.2 |
| 24 | B | 9.2 | 26 | 66 | 1.9 |
| 24 | IT | 0 | 36 | 133 | NA |
| 24 | CT | 15.6 | 28 | 77 | 3.2 |
| 25 | B | 6.9 | 38 | 109 | 1 |
| 25 | IT | 11 | 48 | 121 | NA |
| 25 | CT | 14.2 | 39 | 75 | 1.1 |
| 26 | B | 13.2 | 30 | 51 | 1.1 |
| 26 | IT | 6 | 35 | 97 | NA |
| 26 | CT | 10.4 | 32 | 68 | 1.3 |
| 27 | B | 10.4 | 26 | 54 | 1.1 |
| 27 | IT | 9 | 37 | 58 | NA |
| 27 | CT | 9.8 | 28 | 76 | 1.4 |
| 28 | B | 9.3 | 33 | 53 | 1.4 |
| 28 | IT | 4 | 45 | 56 | NA |
| 28 | CT | 9.7 | 43 | 67 | 0.8 |
| 29 | B | 10.4 | 28 | 49 | 1 |
| 29 | IT | 9 | 36 | 56 | NA |
| 29 | CT | 10.8 | 27 | 74 | 1.3 |
| 30 | B | 18.5 | 32 | 96 | 1.4 |
| 30 | IT | 8 | 69 | 73 | NA |
| 30 | CT | 28.2 | 48 | 71 | 1.3 |
| 31 | B | 8.6 | 32 | 74 | 0.8 |
| 31 | IT | 9 | 38 | 128 | NA |
| 31 | CT | 10.4 | 34 | 62 | 0.6 |
| 32 | B | 12.2 | 31 | 51 | 1.1 |
| 32 | IT | 6 | 35 | 93 | NA |
| 32 | CT | 8.7 | 30 | 72 | 1.2 |
| 33 | B | 13.6 | 25 | 84 | 1.2 |
| 33 | IT | 5 | 29 | 118 | NA |
| 33 | CT | 9.7 | 26 | 76 | 1.3 |
|  |  |  |  |  |  |
